# Relationships among Development, Growth, Body Size, Reproduction, Aging, and Longevity – Trade-Offs and Pace-Of-Life

**DOI:** 10.1134/S0006297923110020

**Published:** 2023-11

**Authors:** Rong Yuan, Erin Hascup, Kevin Hascup, Andrzej Bartke

**Affiliations:** 1 Southern Illinois University School of Medicine, Department of Internal Medicine, 19628 Springfield, Illinois, USA; 2 Southern Illinois University School of Medicine, Department of Medical, Microbial, Cellular Immunology and Biology, 19628 Springfield, Illinois, USA; 3 Department of Neurology, Dale and Deborah Smith Center for Alzheimer’s Research and Treatment, Neuroscience Institute, Southern Illinois University School of Medicine, Springfield, Illinois, USA

**Keywords:** aging, longevity, pace-of-life, trade-offs, developmental programming, growth, reproduction, body size

## Abstract

Relationships of growth, metabolism, reproduction, and body size to the biological process of aging and longevity have been studied for decades and various unifying “theories of aging” have been proposed to account for the observed associations. In general, fast development, early sexual maturation leading to early reproductive effort, as well as production of many offspring, have been linked to shorter lifespans. The relationship of adult body size to longevity includes a remarkable contrast between the positive correlation in comparisons between different species and the negative correlation seen in comparisons of individuals within the same species. We now propose that longevity and presumably also the rate of aging are related to the “pace-of-life.” A slow pace-of-life including slow growth, late sexual maturation, and a small number of offspring, predicts slow aging and long life. The fast pace of life (rapid growth, early sexual maturation, and major reproductive effort) is associated with faster aging and shorter life, presumably due to underlying trade-offs. The proposed relationships between the pace-of-life and longevity apply to both inter- and intra-species comparisons as well as to dietary, genetic, and pharmacological interventions that extend life and to evidence for early life programming of the trajectory of aging. Although available evidence suggests the causality of at least some of these associations, much further work will be needed to verify this interpretation and to identify mechanisms that are responsible.

## INTRODUCTION

Aging is a part of the life course of nearly all living organisms, including all human beings. This is certainly common knowledge, and it often leads to a common belief that there is nothing we can do about aging. Scientific evidence that the biological process of aging can be modified and that these modifications can promote health and extend life has been available for decades, but it is only recently that this evidence is slowly starting to be incorporated into the theoretical framework and daily practice of medicine and into the public health policies.

In this context, we believe that it is timely to consider the trade-offs between development and aging, which are associated with anti-aging interventions, and to identify the underpinning mechanisms. Groundbreaking studies of the effects of calorie restriction (CR, reduced intake of nutrients without malnutrition) in laboratory rodents demonstrated that extension of longevity was associated with reduced body size and suppression of fertility. When juvenile animals were exposed to CR, their growth rate was reduced and sexual maturation was delayed. Severe CR can completely suppress reproductive function while still producing marked benefits in terms of health and longevity. These and other findings lead to the question of whether differences in longevity between different species, as well as between individuals of the same species, are associated with (and perhaps due to) similar trade-offs. In this article, we will briefly review the relationships between sexual maturation and reproduction, as well as somatic growth and adult body size, and longevity. Additionally, we will discuss the role of growth hormone (GH) and insulin-like growth factor-1 (IGF-1), key regulators of growth, maturation, and adult body size, in the control of mammalian aging and the relationships between the “pace-of-life”, the biological process of aging, and its developmental programming. Finally, we will also discuss some of the cellular and molecular mechanisms that may explain the observed associations and trade-offs.

## BODY SIZE AND LONGEVITY: BIG MICE DIE YOUNG, BUT LARGER MAMMALS LIVE MUCH LONGER THAN MICE

The relationship between adult body size and longevity in mammals is very complex. In general, larger species live longer, with the huge bowhead whale being the longest-living mammal. The positive correlation between adult body size and maximal lifespan across all mammalian species is well documented and striking [1]. However, there are important exceptions. Many species of bats live much longer than similarly sized rodents or shrews [2]. Another intriguing example of extended longevity is the naked mole rat (*Heterocephalus glaber*). Despite having a similar body size to laboratory mice, naked mole rats live about ten times longer in controlled laboratory environments [3]. Among mammals, primates live longer than their body size would predict, with our own species being the most extreme example. The average life expectancy of humans far exceeds that of much larger ruminants or equids and the documented maximal human lifespan, 122 years [4], exceeds the longevity of elephants.

The long life of large animals has been linked to reduced risk of extrinsic mortality, particularly mortality due to predation. Longer life expectancy is coupled with the evolutionary emergence of physiological characteristics and reproductive strategies that favor (or are compatible with) long survival. We will discuss these associations later in this article. Mechanistically, extremes of longevity have been linked to molecular and cellular mechanisms of protection from cancer (elephants) [5] and to the capacity to repair DNA damage (bowhead whales) [6]. Extended longevity of bats, naked mole rats, and primates has been related to reduced risk of predation in animals capable of flying or living underground, and protection from multiple causes of extrinsic mortality by social organization combined with intelligence [2, 7]. In addition, the exceptionally long lifespan of naked mole rats has been attributed not only to their specialized underground lifestyle but also to the accompanied remarkable ability to tolerate hypoxic conditions and thrive in environments with limited oxygen availability which may result in reduced oxidative damage [8].

In sharp contrast to the positive correlation of body size and longevity across mammalian species, there are numerous examples of smaller, rather than larger individuals, within the same species having longevity advantage [9, 10]. This is particularly striking and well-documented in laboratory populations of house mice and in domestic dogs [11, 12]. Comparisons of the longevity of mice from different inbred strains or lines selected for differences in growth rate and/or adult body size [13-15] and comparisons of individual mice from a genetically heterogeneous population [11] consistently show the negative correlation between adult body size and longevity. Moreover, mutations producing dwarfism also cause a major extension of longevity. Mice with homozygous alleles of these mutations can survive as much as 50% longer than genetically normal (“wild type”) animals born in the same litter and maintained under identical nutritional and environmental conditions [16, 17].

Comparisons of different breeds of domestic dogs or individual dogs differing in size consistently show a negative correlation between adult body weight and longevity [12]. Very small dogs typically live over 15 years, while dogs from the largest breeds are not likely to reach the age of 10 years [10, 18]. Similar negative associations of body size and lifespan have been described in laboratory rats [19] and in domesticated horses [20], as well as in various human populations [21]. Additionally, studies have reported that interventions extending longevity, such as rapamycin treatment implemented at an early age, are accompanied by reductions in body size [22, 23]. However, the interpretation of the data concerning the relationship of human stature to longevity is complicated by the role of socio-economic status, early life nutrition, access to medical care, progress in medicine, and a plethora of public health policies. Consequently, the existence of a negative correlation between human stature and life expectancy is not universally accepted and is often viewed as controversial [24]. We have discussed this issue in more detail in previous publications [25, 26].

It deserves emphasis that the relationship of the rate of growth and development (a key element of the pace-of-life) to aging and longevity differs in important ways from the association of adult body size with the same life history traits. Thus, slower growth is related to slower aging and longer life in comparisons both between and within species, but smaller adult size predicts longer life only in comparisons between individuals from the same species. The paradox of the opposite relationships is not easily explained. One study suggested that the circulating levels of IGF-1, the key mediator of stimulatory action of pituitary GH on somatic growth, are unexpectedly lower in larger than in smaller species [27]. This contrasts with the positive correlation between IGF-1 levels and body size within a species [28]. As will be discussed later in this article, we favor a different explanation, namely the role of the rate of growth, development, and maturation (combined with the duration of growth and with other phenotypic characteristics) in the programming of aging and longevity. Naturally, these explanations are not mutually exclusive and other mechanisms may also be involved. For example, disparities in longevity both within and between species could be attributed to factors such as cell division dynamics and growth [29], the composition of cell membranes [30], DNA damage and repair [31], and the erosion of telomeres and telomerase activity [32]. These mechanisms likely interact with one another. Additionally, it is plausible that the correlations between body size and longevity do not imply a cause-and-effect relationship. Instead, both factors could be influenced by a shared factor, potentially one of the “pillars” or “hallmarks” of aging, as discussed in the antagonistic pleiotropic theory [33] or the hyperfunction theory [34].

A slow pace of growth may also shape aging and lifespan *via* its impacts on brain development [35]. A previous study analyzed 493 mammal species, spanning rodents to cetaceans, revealing a compelling link: mammals with larger brains relative to their body size tend to have longer lifespans and extended reproductive periods [36]. This observation is intriguing, given that larger brains come with metabolic expenses and protracted developmental phases. Natural selection, therefore, should favor the evolution of larger brains only if they offer compensatory advantages. One such advantage is the facilitation of adaptive responses to novel or complex socioecological challenges, a concept encapsulated by the Cognitive Buffer Hypothesis (CBH) [37]. CBH posits that a larger brain enhances behavioral adaptability in response to changing environmental conditions, simplifies the learning process, and equips species to surmount ecological hurdles effectively. Additionally, this adaptability promotes the formation of stable social groups, aligning with the Social Intelligence Hypothesis (SIH), which suggests that species living in such groups face increased cognitive demands, thus necessitating larger brains to cope with the intricacies of group living [38]. Indeed, stable social groups offer several advantages, including cooperative defense, resource sharing, shared parental care, and improved reproductive success, enhancing survival for individuals and offspring. Interestingly, social structure can play a decisive role in shaping the relationships between body size, reproduction, and lifespan. This is evident in eusocial insects like ants and bees, as well as mammals like the mole rat. In these species, reproducing females often exhibit significantly larger body sizes yet live considerably longer than other members of their societies. Thus, in these animals, increased reproductive effort is associated with extended rather than reduced longevity.

## HORMONAL CONTROL OF GROWTH AND LONGEVITY: ROLE OF PITUITARY GROWTH HORMONE IN THE CONTROL OF MAMMALIAN AGING

It is now more than 25 years since we suggested that GH is importantly involved in the control of mammalian aging. This was based on studies in GH transgenic mice and in mice with hereditary dwarfism. We reported that giant transgenic mice have numerous phenotypic characteristics that resemble aging [39] and confirmed that these animals live much shorter than their normal siblings [40]. We also reported that the longevity of Prop1^df^ (Ames dwarf) mice, which are deficient in several adenohypophyseal hormones including GH, is greatly extended [17]. These findings implied that normal levels of GH signaling incur “costs” in terms of longevity and that pathological access to GH leads to accelerated aging. The evidence for the role of GH in the control of aging was greatly strengthened by subsequent demonstration that isolated GH deficiency caused by deletion of the key hypothalamic regulator of GH biosynthesis and secretion, GH releasing hormone (GHRH) [41], or by a mutation of the GHRH receptor (GHRHR) gene [42], as well as deletion of the GH receptor (GHR) gene [43], also produce an impressive extension of mouse longevity. In other words, the blockade of GH production or its actions is sufficient to increase life expectancy. These studies also indicated that extension of life in the absence of GH signals is a highly reproducible finding and that it is not limited to a particular mutation, the genetic background of the animals, or husbandry conditions in one laboratory. This point merits emphasis, since a reduction rather than an extension of the longevity of hereditary dwarf mice was seen in one older study [44], an observation now believed to be due to husbandry conditions and animal health issues in this laboratory [45-47].

Importantly, additional studies in Ames dwarf mice provided evidence that the link between GH deficiency and the long life of these animals represents a cause: effect relationship. Six weeks of GH replacement therapy, started at one or two weeks of age, significantly reduced the longevity of these animals [48]. Moreover, most of the phenotypic characteristics that distinguish Ames dwarf from normal (wild type) mice and are believed to represent mechanisms and/or markers of their slower aging and extended longevity were completely or near-completely normalized (“rescued”) by this regimen of GH replacement [48-50]. The mechanisms by which early-life GH treatment alters adult phenotype and longevity are almost certainly epigenetic and involve histone modifications [51].

The dramatic impact of GH deficiency, GH resistance, and GH excess on the longevity of laboratory mice prompted many questions about the applicability of these findings to other species, particularly to humans [52]. Pathological GH excess in the syndromes of acromegaly and gigantism reduces life expectancy in humans [53, 54], thus echoing the findings in giant GH transgenic mice [55, 56]. Moreover, many symptoms of these conditions, including increased risk of diabetes, cardiovascular disease, and some types of cancer, resemble symptoms of normal aging. However, in contrast to the findings in various types of dwarf mice, humans with isolated GH deficiency (IGHD) or GH resistance (Laron syndrome) do not live longer [57, 58] and reduced life expectancy was reported in one population of individuals with hereditary IGHD [59]. Interestingly, even though life expectancy is not extended by genetic syndromes that block GH signaling, GH-resistant, and GH-deficient individuals are significantly protected from chronic aging-associated diseases including cancer, diabetes, and atherosclerosis, show improvements in the maintenance of various physiological functions into advanced age [60], and in at least one population of IGHD individuals appear to be more likely to achieve exceptional longevity [61]. In terms of average longevity, these protective features of GH-deficient and GH-resistant individuals appear to be counterbalanced by an increased risk of early deaths, particularly deaths related to accidents and alcohol abuse [25, 60]. Increased chances of living to very old age were reported also in the “little people of Krk,” a population of individuals with a GH deficiency caused by a mutation of the *Prop1* gene, the same gene which is mutated in the long-lived Ames dwarf mice [57].

Several recent and ongoing studies addressed the possible role of variations in GH and IGF-1 signaling within the normal range in the control of human aging. Offspring of long-lived families identified in the Leiden Longevity Study [62] have been consistently shown to exhibit several favorable health outcomes compared to their partners. This includes a reduced incidence of diabetes, hypertension, and hypercholesterolemia, as well as a decreased risk of other prevalent age-related diseases, such as cardiovascular diseases (e.g., coronary artery disease, heart failure, and stroke), neurodegenerative diseases (e.g., Alzheimer’s disease and Parkinson’s disease), osteoporosis, chronic obstructive pulmonary disease (COPD), arthritis, age-related macular degeneration, renal diseases, age-related hearing loss, and other conditions commonly associated with aging [63-68]. Importantly, the offspring of long-lived individuals also demonstrate a lower mortality rate compared to their partners [69]. It was also reported that these individuals have reduced rates and “tighter” control of GH secretion [62, 70]. These data suggest that a normal level of GH signaling acts to accelerate aging in humans as it does in laboratory stocks of house mice and that lower levels of GH are beneficial for healthspan and lifespan. Of course, it is difficult to exclude alternative interpretations, such as the existence of co-regulators that influence both GH secretion and the aging process.

Many studies addressed the possible involvement of IGF-1 as a part of the IGF/insulin signaling (IIS) in human aging, and both positive and negative associations with longevity have been reported. While a detailed analysis of this complex set of observations is outside the scope of this brief article, we would like to point out that a likely reason for the discrepancies in findings from different studies was recently proposed by Zhang and colleagues in a study that analyzed the levels of IGF-1, mortality, and aging-related diseases, including dementia, diabetes, cancer, vascular disease, and osteoporosis, among more than 400,000 people aged from the 30s to the 70s [71]. The results suggest that IGF-1 is a nonlinear predictor of risk, and interacts with age to modify risk for a variety of clinical events. Specifically, younger individuals with high IGF-1 levels exhibited a protective effect against the disease, whereas older individuals with elevated IGF-1 levels were at an increased risk of developing an incident disease or experiencing mortality [71]. Furthermore, the association between IGF-1 levels and disease risk displayed a U-shaped relationship, indicating that excessively high and low levels of IGF1 may be detrimental to disease susceptibility [71].

The intricate effects of IGF1 on aging, including genetic variations, sex disparities, and the interaction between IGF1 and age, were examined in a comprehensive meta-analysis, using 32 mouse inbred strains [72-74]. These strains represent the major diversity in the genome of *Mus musculus,* providing a rich source of genetic variation, which is one to two orders of magnitude higher than the level of sequence diversity observed in human populations [75]. The results revealed an association between lower IGF-1 levels and extended median lifespan across the inbred strains [72]. However, a sex-specific correlation was observed between IGF1 levels and the lifespan variation [74]. In females, lower IGF-1 levels were associated with an increased risk of death at a young age (<180 days), but an extended maximum lifespan, leading to greater variation in lifespan. In males, although no significant alteration in the risk of early death was observed, higher IGF-1 levels were associated with an extended maximum lifespan. Therefore, in males, the higher IGF-1 level is associated with elevated variation in the lifespan [74]. Notably, an intervention study was conducted on female and male mice at old age (18 months) using the IGF-1 receptor (IGF1R) antibody. This treatment improved female healthspan and resulted in a 9% increase in median lifespan, along with reductions in neoplasms and inflammation. However, no significant changes were observed in male mice [76]. These findings further highlight the sex-specific effects of IGF-1 on aging and underscore the importance of considering the time window for targeting IGF-1 signaling to extend lifespan. Particularly, it raises intriguing questions about whether increasing IGF-1 levels during old age could potentially extend the maximum lifespan of males.

## REPRODUCTIVE DEVELOPMENT, AGE OF SEXUAL MATURATION, AND AGING. TRADE-OFFS AND DIFFERENT LIFE HISTORIES

Comparisons of the reproductive effort in short- and long-lived species of mammals reveal major differences and suggest the existence of trade-offs. Short-lived mammals typically have an early age of puberty, short gestation, large litters, and early age of weaning associated with short intervals between the litters. For example, mice and other small rodents can reach sexual maturity in less than two months and produce litters as large as 10-12 pups at intervals of one to two months. In contrast, large, longer-living species generally exhibit opposite reproductive characteristics: late puberty, single offspring or very small litters, and a long period of nursing. For example, ruminants or equids (including domestic cows, sheep, and horses) may not reach sexual maturation in the year they are born, have gestation lengths ranging from several to 11 months, produce one, or, less commonly, two offspring once a year, and nurse them for several months. Domestic dogs exhibit very large differences in longevity between small and large breeds and between mixed breed animals differing in body size (details and references earlier in this article). Bargas-Gallaraga and colleagues recently reported that reproductive investment negatively impacts longevity in this species and that this relationship could not be explained by the correlation of both reproductive effort and longevity to body size [77]. A remarkable study of life history trajectories in over seven thousand female southern elephant seals in their natural habitat revealed a correlation between the age at first reproduction and the onset of actuarial senescence (defined as age-related increase in mortality) [78]. However, early breeders in this population outperformed delayed breeders in terms of survival and net reproductive output [78]. Apparently, differences in the individual ability to cope with early life challenges can override the effects of trade-offs between reproductive performance and longevity.

Evidence for trade-offs between reproduction and longevity was derived from studies of calorie restriction (CR) [79] and from studies in which the macronutrient composition of the diet (primarily the relative content of protein and carbohydrate) was varied by the investigators [80, 81] or determined by the choices of experimental animals themselves [82, 83]. Generally, a higher intake of calories and protein favored reproduction but not survival [83]. In laboratory rodents, CR can significantly extend longevity in most of the examined populations and can block or delay puberty and lead to infertility or to reduced litter size and increased intervals between the litters [84]. However, these trade-offs were nuanced and depended on the specific features of the study. Specific outcomes depend on the species (for example rats *vs*. mice), age of onset of CR, and the percentage of reduction in food intake [84]. In addition to the various suppressive effects on reproduction, CR was also reported to delay reproductive aging and to increase the age at which reproduction can be “re-awakened” by refeeding [85]. Delayed puberty, reduced litter size and increased intervals between litters as well as various indications of delayed reproductive aging were reported also in genetically dwarf mice in association with a remarkable extension of longevity [86].

The relationship of the age of sexual maturation to aging and longevity deserves particular emphasis. The association of earlier puberty with shorter lifespan is seen in comparisons both between and within species. Importantly, the age of sexual maturation in short-living animals is early not only in terms of chronological age but also in proportion to the average life expectancy. For example, laboratory mice reach puberty at the age of three to six weeks which represents roughly 3-5% of their average longevity, while humans typically reach puberty at 12-15 years of age, corresponding to 15-19% of the average lifespan. Correlation between the age of puberty and longevity also was detected in comparisons of individuals or cohorts (such as different strains or breeds) within the same species [73].

Given the robust correlation observed between the age of sexual maturation and lifespan, and the intricate connection between body size with aging and longevity, an intriguing question emerges: Can retarding reproductive development while leaving somatic growth unaffected lead to delayed aging and extended lifespan? Addressing this question is challenging due to the tightly coordinated control between somatic growth and reproductive development. Interestingly, our recent study involving heterogeneous UM-HET3 mice, where metformin was administered during early life (15-56 days), found that while metformin increased circulating IGF1 levels and body size, it significantly delayed the age of female sexual maturation [87]. This implies that metformin treatment could potentially decouple the regulatory pathways controlling somatic and reproductive development. The underlying mechanism of this effect may be linked to the molecular function of metformin in upregulating AMPK activity [88]. Mammalian reproduction is an energy-consuming process that occurs when there is adequate nutrition [89]. AMPK is a sensor of nutrient status, and it is activated by the decrease of ATP/AMP ratio or starvation. Activated AMPK acts to switch off ATP-consuming pathways, such as protein synthesis, lipogenesis, and gluconeogenesis, and turn on ATP-generating pathways such as fatty acid oxidation, glycolysis, and autophagy [90]. On the molecular level, activated AMPK inhibits the mammalian target of rapamycin (mTOR) by directly phosphorylating the tumor suppressor tuberous sclerosis complex 2 (TSC2) and regulatory-associated protein of mTOR (RAPTOR) [91]. Elevating mTOR signaling can significantly accelerate female sexual maturation and enhance female reproduction [92]. Importantly, in recent studies, suppression of mTOR by rapamycin was shown to suppress reproduction but extend healthspan and lifespan [22, 23]. The complex relationships among reproduction, body size, and aging are far from being resolved, and understanding the roles of AMPK and mTOR signaling in the co-regulation of development and aging is of great interest. For a discussion of the underlying evolutionary processes and a recent overview of this field of study please see [93-95].

## EARLY LIFE PROGRAMMING OF ADULT CHARACTERISTICS. CAN SLOWING THE PACE-OF-LIFE PRODUCE LONGEVITY BENEFITS?

Interest in the role of early life events in programming development started at least 64 years ago with the pioneering work of Waddington on the “canalization” of developmental events [96]. These studies provided the groundwork for the present understanding of epigenetic phenomena and mechanisms of induction of phenocopies by environmental factors. Studies of the effects of starvation during pregnancy on the risk of cardiovascular and metabolic disease in adult offspring lead to the now solidly grounded concept of the Developmental Origins of Health and Disease (DOHaD) [97]. While most studies related to DOHaD addressed detrimental effects of starvation, malnutrition, toxicants, and stress during pregnancy, there is also considerable evidence that environmental insults during childhood and adolescence can have lasting effects on health and the risk of chronic disease [98, 99]. Based on these findings, it would seem reasonable to suggest that early life events can shape the pace-of-life and associated trade-offs such as the partitioning of available resources between reproduction and processes promoting longevity.

Our studies of the effects of GH replacement therapy in GH-deficient long-lived Ames dwarf mice provided additional support for early life programming of aging and longevity [48]. In this work, dwarf animals were injected with GH for only six weeks starting at one or two weeks of age. This produced the expected acceleration of growth during the period of treatment and an increase of adult body weight to values approximately intermediate between the weight of control (vehicle injected) dwarfs and their normal (wild type) siblings. As mentioned earlier in this article, various phenotypic characteristics related to the “hallmarks” (“pillars”) of aging, including markers of brain gliosis, plasma insulin, adiponectin, ketone bodies, and lipid profiles, measured approximately one year or even later after the end of GH therapy, were completely (or near completely) normalized so that they no longer differed from values measured in saline-treated wild-type controls [48-50]. Moreover, the remarkable longevity of these mice was significantly reduced by six weeks of GH treatment in early life [48]. Apparently, this early-life endocrine intervention produced long-lasting, likely permanent physiological changes reflecting (and/or contributing to) profound alterations in the trajectory of aging. The underpinning mechanisms were almost certainly epigenetic and evidence available to date suggests that they involved histone modifications [51].

## PACE-OF-LIFE AND AGING. RELATIONSHIPS OF EARLY GROWTH AND EARLY REPRODUCTIVE EFFORT TO LONGEVITY

In previous sections of this article, we have discussed the evidence for the role of trade-offs with growth, maturation, and reproduction in the control of aging and the involvement of GH and IGF-1 in mediating these trade-offs. Also mentioned were paradoxical differences between relationships of adult body size to longevity in comparisons among vs. within species. The picture that emerges from the available evidence is consistent with the concept of developmental programming of aging which is supported by much experimental evidence as well as theoretical considerations [96, 100-104]. In particular, faster early growth and greater body weight of juveniles have been related to higher morbidity and mortality in studies of mice [105], dogs [106], and humans [107, 108]. In laboratory mice, interventions that affect early somatic growth were shown to have a major impact on longevity. Shindyapina and her colleagues have shown that treatment of genetically diverse UMHET3 mice with rapamycin during the first 45 days of postnatal life resulted in reduced growth rate and extended longevity (median life span) and importantly, also healthspan [22]. As was mentioned earlier, a relatively brief period of GH treatment in early life accelerated growth and reduced longevity in Ames dwarf mice [48]. Extension of longevity by early life treatment with rapamycin was achieved also in a small (planktonic) crustacean, Daphnia, and in a fruit fly, Drosophila [22, 23]. Similar to early growth rate, early sexual maturation was negatively related to longevity in various studies [73].

What we believe deserves particular emphasis is that the reciprocal relationship between longevity and key elements of the pace-of-life, namely growth, maturation, and reproductive effort, apply to comparisons between different species of mammals as well as between different strains, breeds, or individuals with a species. This is in marked contrast to the relationship of longevity to adult body size, which is opposite rather than consistent in these comparisons. It is interesting that differences in the pace of life also correlate with the differences in life expectancy among humans with extremely short stature. Thus, short stature due to isolated GH deficiency in the Itabaianinha cohort or to GH resistance is associated with a slow pace-of-life (slow growth, delayed puberty, and reduced fecundity), protection from various age-related diseases and conditions leading to “healthy aging” as well as with normal life expectancy [25, 109], while short stature in various populations of pygmies is associated with fast pace-of-life (fast development and early age of first pregnancy) and very short life [110]. A particularly striking example of a reciprocal relationship between the pace of life and longevity is provided by an African fish, *Nothobranchius furzeri* which lives in ephemeral ponds. The eggs of these remarkable animals survive in the soil after the ponds dry up and hatch when the pond is re-established by the next rainy season. The young fish develop at the rate that has been described as “explosive”, reproduce and age rapidly resulting in the average longevity of four to six months, the shortest lifespan of any vertebrate [111]. Importantly, rapid aging and early death are seen also in captive fish of this species which are maintained in aquaria that (of course) do not seasonally dry up and are provided with a reliable supply of food [112], indicating the genetic accommodation of the species to its natural environment.

The definition of the pace-of-life generally includes basal metabolic rate and slow metabolism was frequently implicated in delayed aging, however, longevity is not simply determined by the rate of metabolism. The most striking example of this is provided by comparisons between mammals and birds. Birds have higher metabolic rate than mammals of the same size and yet live longer rather than shorter. This may be due to reduced extrinsic mortality of organisms that can avoid predators and other environmental risks by flying [113, 114]. Interestingly, the average daily metabolic rate measured by oxygen consumption per unit body mass is increased rather than reduced in long-lived genetically growth hormone deficient or growth hormone resistant mice [115]. Analysis and interpretation of data on energy metabolism is complicated by differences between basal metabolic rate, resting metabolic rate, and energy expenditure or field metabolic rate, and by different ways of reporting bioenergetic data. We have dealt with these complex issues in earlier publications [116-118].

Unraveling the trade-offs among body size, reproduction, aging, and longevity requires further research to understand the underlying mechanisms and their implications for human health and aging. Incorporating this knowledge into medical practice and public health policies has the potential to promote health and extend lifespan in the future.

## Abbreviations:

DOHaD: Developmental Origins of Health and Disease
GH: growth hormone
GHR: growth hormone receptor
GHRH: growth hormone releasing hormone
IGF-1: insulin-like growth factor-1
IGHD: isolated growth hormone deficiency
